# Prairie management practices influence biodiversity, productivity and surface–atmosphere feedbacks

**DOI:** 10.1111/nph.70195

**Published:** 2025-05-14

**Authors:** Ran Wang, John A. Gamon, Katharine F. E. Hogan, P. Roxanne Kellar, David A. Wedin

**Affiliations:** ^1^ School of Natural Resources University of Nebraska‐Lincoln Lincoln NE 68583 USA; ^2^ Lauritzen Gardens Omaha NE 68108 USA; ^3^ Department of Biology University of Nebraska Omaha Omaha NE 68182 USA

**Keywords:** controlled burning, grassland, imaging spectroscopy, prescribed fire, restoration, thermal remote sensing

## Abstract

- Grassland restoration efforts aim to reestablish vegetation cover and maintain ecosystem services. However, there is a lack of systematic evaluation of the effects of grassland restoration and management strategies on biodiversity, productivity and surface–atmosphere feedbacks affecting climate.
- Through a multiyear grassland restoration experiment in a tallgrass prairie site in Nebraska, USA, we investigated how different management practices affected biodiversity, productivity and surface–atmosphere feedbacks using a combination of *in situ* measurements and airborne hyperspectral and thermal remote sensing.
- Our findings indicated that management treatments affected vegetation diversity, productivity and energy balance. Higher diversity plots had higher plant growth, albedo, canopy water content and lower surface temperature, indicating clear effects of management treatments on grassland ecosystem processes influencing surface–atmosphere feedbacks of mass and energy.
- The coherent responses of multiple airborne remote sensing indices illustrate potential cobenefits of grassland restoration practices that enhance ecosystem productivity and biodiversity and mitigate climate change through surface–atmosphere feedbacks, offering a new strategy to address the challenges of biodiversity loss and climate change in grassland ecosystems.

## Introduction

Grasslands constitute *c*. 40% of the Earth's terrestrial area and cover a wide range of geographic locations (White *et al*., 2000). Besides supporting agricultural production through grazing, well‐maintained grasslands sustain a variety of plant and animal species, stabilize soil and prevent erosion, improve water retention and groundwater recharge and provide habitat and food sources for pollinators (Murphy *et al*., 2016; Bengtsson *et al*., 2019). Grasslands can also be important soil carbon sinks and store approximately one‐third of global terrestrial carbon, mostly in the form of biomass and soil organic carbon (Bai & Cotrufo, 2022). However, grasslands have experienced a world‐wide decline during the last century, leaving half of grasslands degraded (Bardgett *et al*., 2021) with few intact grasslands remaining (Scholtz & Twidwell, 2022). Grassland degradation is often caused by landcover conversion to crops and forestry, land abandonment, overgrazing, woody encroachment and species invasion (Parr *et al*., 2014; Bardgett *et al*., 2021). Abandonment of historical indigenous management practices (e.g. frequent fire) has also altered grassland composition and function (Pyne, 2019). Altered disturbance regimes (e.g. grazing intensity and timing and frequency of fire) and species reintroduction (e.g. seeding) can gradually restore grassland aboveground diversity and function and belowground structure (Buisson *et al*., 2022), suggesting that effective restoration strategies may have multiple cobenefits.

Nature‐based solutions aim to adapt to and mitigate climate change effects while improving sustainable livelihoods, protecting natural ecosystems and biodiversity, and preventing the degradation and loss of natural ecosystems (IUCN, 2016). Restoring grassland cover and biodiversity can serve as an effective strategy for mitigating negative impacts of global climate change by facilitating relatively fast and resilient carbon sequestration (Griscom *et al*., 2017; Fargione *et al*., 2018; Bai & Cotrufo, 2022). Conservation programs, such as the United States Department of Agriculture (USDA) Conservation Reserve Program (CRP) (Allen & Vandever, 2003; Hellerstein, 2017) and British Coronation Meadows (Thomas, 2021), aim to reestablish valuable land cover to help improve water quality, prevent soil erosion, enhance carbon sequestration, reduce nitrogen runoff and provide healthy habitat for wildlife and pollinators. Despite reports of successfully achieving these goals individually (Gelfand *et al*., 2011; Gleason *et al*., 2011; Becker *et al*., 2022), there is a lack of systematic evaluation of the combined effects on biodiversity, ecosystem function and climate feedbacks of grassland restoration practices, especially in conjunction with heterogeneous management processes such as prescribed burning, mowing and grazing. This leaves a gap in our understanding about how management and restoration practices contribute to critical ecosystem processes that may offer climate benefits.

Terrestrial surface energy balance refers to the equilibrium between incoming solar radiation, energy stored in soil and plant biomass, and outgoing energy in the form of energy reflected (albedo) and energy used for heat and evapotranspiration, typically measured as surface temperature (Schulze *et al*., 2019; Still *et al*., 2019). Along with mass balance, understanding this energy balance is essential for managing ecosystems, predicting their responses to climate change and assessing their role in carbon and water cycles (Bonan, 2008). Emerging remote sensing methods can capture states and processes related to mass and energy exchange (Hall *et al*., 1992), but applying these methods to prairie restoration options remains largely underexplored (Blackburn *et al*., 2021). One reason is the scale mismatches between common spaceborne remote sensing methods and those of land ownership and management units such as individual fields or pastures that can be readily sampled. Another reason is the complex, varying management practices themselves, which often vary from year to year or from one landowner to the next. Airborne remote sensing, with the combined power of explicit spectral and thermal sampling at fine spatial resolutions, readily matches the scale of typical grassland management practices and offers a powerful set of tools for evaluating the effects of grassland restoration among a range of management treatments.

Management practices affect grassland biodiversity and productivity (Knapp *et al*., 1999; Silvertown *et al*., 2006; Fuhlendorf *et al*., 2009; Hovick *et al*., 2015) and can further impact surface climate feedbacks by regulating energy and water cycles (Mahecha *et al*., 2024; Miralles *et al*., 2025). For instance, positive productivity and biodiversity relationships have often been reported in natural or low‐input grassland ecosystems (Tilman *et al*., 2001; Isbell *et al*., 2009) while intensively managed low‐diversity grasslands (e.g. monocultures with added fertilizer or irrigation) can have higher productivity than high‐diversity communities (Silvertown *et al*., 2006; Johnson *et al*., 2010; Brun *et al*., 2019). In terms of pyric herbivory (Fuhlendorf *et al*., 2009), fire can remove aboveground plant biomass, increase soil nutrient availability because of higher mineralization and fixation rates (Skidmore *et al*., 2010; McMillan *et al*., 2023) and stimulate germination of certain species that may otherwise lie dormant (Ramos *et al*., 2019). On the other hand, fire suppression may lead to higher aboveground carbon stocks due to woody encroachment but at the cost of biodiversity loss at various trophic levels (Abreu *et al*., 2017). When applied together, fire and grazing are known to create landscapes that are more resilient than those with less complex, homogeneity‐based management practices (Hovick *et al*., 2015). Together, these observations suggest that positive effects of biodiversity, productivity and energy balance could result from proper grassland management. However, it remains a challenge to monitor the response of prairie ecosystems to different management practices (e.g. fire and grazing) at large spatial and temporal scales, in part due to the large expanse and remote location of many grassland sites. Herbivore movement and uneven fire burn patterns add further complexity to these topics (Rossi *et al*., 2024).

To address these topics, we chose to evaluate the influence of grassland management treatments in an experimental setting. In this study, we conducted a multiyear experiment in a tallgrass prairie restoration site near Lincoln, Nebraska, USA, with various management treatments that included contrasting seeding diversity, burning and haying practices. We hypothesized that the interconnected linkages between productivity, biodiversity and surface–atmosphere feedbacks in grassland ecosystems are influenced by management practices. We propose that certain management practices can enhance grasslands biodiversity and productivity in low‐input settings (e.g. without herbicide application after the initial experiment setup), which further affect the atmosphere and climate through surface–atmosphere exchanges of energy and matter. Using airborne hyperspectral and thermal remote sensing, our primary goal was to investigate the impact of several common management strategies on grassland biodiversity (species and functional composition) and ecosystem function (productivity and energy balance) with implications for nature‐based solutions to address climate mitigation.

## Materials and Methods

### Field site description and experimental design

This study was conducted at the Bobcat Restoration Experiment (40.71°N, 96.82°W), a *c*. 12‐ha field located in Lancaster County, Nebraska, that has been annually hayed for several decades, partially under USDA CRP contract management. Before the restoration experiment, the site was dominated by invasive smooth brome (*Bromus inermis* Leyss.) and native Indiangrass (*Sorghastrum nutans* (L.) Nash). The average growing season temperature (May to September) is 28°C, and the average annual precipitation is *c*. 74 cm.

**Fig. 1:**
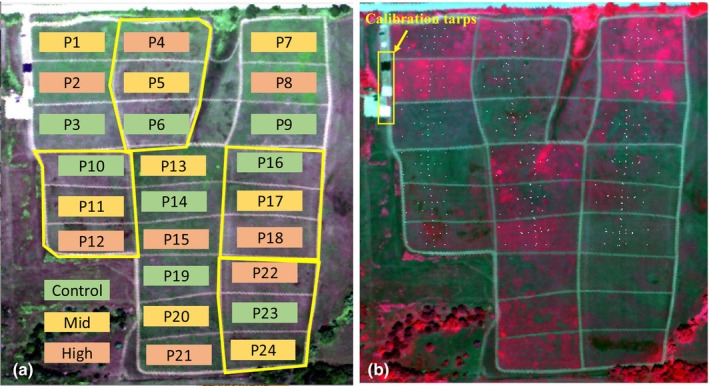
Experimental design of the Bobcat Restoration Experiment. (a) Schematic of the plot locations, seeding treatments (control, mid‐ or high‐diversity) and management (burning and haying) laid on top of the airborne true color reflectance image (red: 680 nm, green: 550 nm and blue: 460 nm). Green plots are control, yellow plots are mid‐diversity and orange plots are high‐diversity. The yellow boundaries indicate blocks that received annual haying since 2019. The rest of the plots were burned on 10 May 2022. (b) Locations of the 2022 field vegetation sampling (white dots) on top of the airborne false color reflectance image (red: 800 nm, green: 680 nm and blue: 550 nm). Also shown are the locations of calibration tarps (black, gray and white squares within the yellow rectangle). Airborne imagery was collected using the Kestrel imaging spectrometer on 20 July 2022.

We arranged the treatments into eight blocks of three plots each (24 plots in total; Fig. 1a; Table 1) to evenly distribute seeding treatments and management strategies (haying vs burning) while accounting for site heterogeneity in soils and extant vegetation. The size of each plot was *c*. 0.40 ha (Fig. 1a). The seeding treatments included the following: (1) a high‐diversity local ecotype (‘high‐diversity’) seed mix of 154 species from Prairie Plains Resource Institute in Aurora, Nebraska, (2) a local ecotype modified CRP‐style pollinator (‘mid‐diversity’) mix of 40 species from Prairie Legacy Inc., Nebraska, and (3) a low‐diversity control group with no seed applications. For the high‐ and mid‐diversity plots, we applied two glyphosate treatments using a boom sprayer (at the standard industry rate of 0.84 kg/ha) to weaken the preexisting vegetation, mostly smooth brome (*B. inermis*), on 18 November 2018 and 19 April 2019. The high‐diversity mix was broadcast from an all‐terrain vehicle spreader on 29 April 2019 onto a lightly disked surface to ensure seed–soil contact. The mid‐diversity mix was drill‐seeded on 1 May 2019. The control plots received no glyphosate treatments nor seed applications. Management treatments (Fig. 1a) consisted of annual mowing or haying in August starting 2019 or burning on 10 May 2022. The plots assigned to the haying treatment were mowed during the first growing season of the experiment (30 July 2019), as not enough biomass was present for effective baling. Haying commenced the following growing season. The detailed seeding and management treatments are further summarized in Tables 1, 2.

**Table 1:** Summary of management treatments applied to the experimental blocks.

Experimental block	Plots within block	Management treatment of block	Management date(s)
1	1, 2, 3	Prescribed burn	10 May 2022
2	4, 5, 6	Hay/mow	30 July 2019; 10 August 2020; 20 August 2021
3	7, 8, 9	Prescribed burn	10 May 2022
4	10, 11, 12	Hay/mow	30 July 2019; 10 August 2020; 20 August 2021
5	13, 14, 15	Prescribed burn	10 May 2022
6	16, 17, 18	Hay/mow	30 July 2019; 10 August 2020; 20 August 2021
7	19, 20, 21	Prescribed burn	10 May 2022
8	22, 23, 24	Hay/mow	30 July 2019; 10 August 2020; 20 August 2021

**Table 2:** Summary of seeding treatments applied to the plots (na, not applicable).

Diversity level	Glyphosate treatments	Seed mix	Seeding application
Control	na	na	na
Mid	18 November 2018; 19 April 2019	Local ecotype modified CRP‐style pollinator of 40 species	Drilled
High	18 November 2018; 19 April 2019	Local ecotype high‐diversity seed mix of 154 species	Disk and broadcast

### Field species data

Species data were recorded for 18 of the 24 plots (Fig. 1b) in August 2022. Within each plot, we started roughly from the center of the plot and laid out four 15‐m‐long transects in the west–east and north–south directions. We sampled species data at the center of the plot, and at meter 7 and at meter 14 along each transect in the four directions (nine locations per treatment plot). In addition to these transects, we also sampled species data from six subplots (in a 2 × 3 array along the east–west direction; Fig. 1b) within each plot. Combining the transect and subplot design offered the opportunity to capture the spatial pattern of species distribution within each plot and closely match the *in situ* proximal remote sensing sampling and airborne imagery. This design resulted in 15 vegetation sampling positions within each plot. At each sampling location, species percentage cover within a 1‐m^2^ quadrat was recorded. The six subplots were originally designed to be 5 m away from the edge of the plot and roughly 30 m apart from each other. However, the 5‐m margin of the subplots varied over the course of the experiment due to mowing and vehicle traffic around the edges of the plots. To avoid potential edge effects, we removed any data sampled within 5 m from edges of each plot. Using the percentage cover data, we calculated species richness, Shannon's index based on species and Shannon's index based on functional groups (C_3_ grasses, C_4_ grasses and forbs).

### *In situ* proximal remote sensing data

To calibrate airborne data and interpret the treatment effects across the full solar spectral range, we collected ground reflectance data for six plots (P1, P2, P3, P10, P11 and P12; Fig. 1) using a portable spectrometer (PSR+ 3500; Spectral Evolution Inc., Lawrence, MA, USA), which covered the 350–2500 nm spectral range. Measurements were taken at every meter along the four 15‐m north–south and east–west transects, yielding 60 samples for each plot. Spectral radiance was sampled using a 2‐m fiber (Spectral Evolution Inc.) at a height of *c*. 1.5 m above the ground. The field of view (FOV) of the fiber was 25°, and the size of the ground sampling was *c*. 1 m. A calibration panel (Spectralon, Labsphere, North Sutton, NH, USA) was measured before field spectral data collection in each plot. Then, canopy reflectance was calculated as the ratio between radiances of vegetation and the calibration panel. Field proximal remote sensing collection was conducted on 20 July and 21 July 2022 with all the measurements collected between 10 a.m. and 4 p.m. to minimize shadow effects.

### Airborne data collection and processing

Airborne hyperspectral and thermal images were collected on 20 July 2022 (*c*. 13:35 local time) using an imaging spectrometer (AISA Kestrel; Specim, Oulu, Finland) and a thermal imager (IR‐TCM 640; Jenoptik Optical Systems GmbH, Jena, Germany) mounted on a fixed‐wing airplane (Piper Saratoga; Vero Beach, FL, USA). Images were collected from *c*. 1.5 km above the ground level. Air temperature was *c*. 30.6°C at the flight time. Air temperature was measured at the Spring Creek Prairie Site (3.75 km from the field site) within the US Climate Reference Network.

### Hyperspectral data

The Kestrel imaging spectrometer collected visible–near‐infrared (VIS‐NIR) (400–1000 nm) hyperspectral data with a 2.3 nm spectral resolution (full width at half maximum). The FOV is 40°, and the ground pixel size was *c*. 1 m. An inflight GPS and inertial measurement unit (IMU) (RT3000; Oxford Technical Solutions Limited, UK) was used to record the location and rotation attributes of the aircraft, which was then used for image geometric correction. To obtain surface reflectance data by compensating atmospheric absorption and scattering effects, we scanned three (white, silver and black) 9 m × 9 m calibration tarps (Odyssey; Marlen Textiles, New Haven, MO, USA) using the PSR+ field spectrometer. Ground calibration targets were sampled within 30 minutes of the overflight on 20 July 2022. We then incorporated the reflectance of the three calibration tarps with a radiative transfer model (MODTRAN 5.0; Berk *et al*., 2008) using a Bayesian method as previously documented (Wang *et al*., 2021).

To interpret the thermal image (details will be discussed later) and understand metrics related to the ecosystem energy balance, we calculated vegetation indices including NDVI, VIS‐NIR albedo and the Water Band Index (WBI; Peñuelas *et al*., 1993), from the airborne hyperspectral image. Reflectance values at 680 and 800 nm were used as the red and NIR bands to calculate NDVI, which has long been used as an indicator of grassland productivity, radiation absorption (Supporting Information Fig. S1) and biomass (Gamon *et al*., 1995; Wehlage *et al*., 2016; Wang *et al*., 2016a,b). Visible–near‐infrared albedo was calculated as mean reflectance in the 450–900 nm spectral range. The WBI was calculated as the ratio between reflectance at 900 and 970 nm. The WBI has long been used as a measure of canopy water content and yields a similar result to equivalent water thickness (Gamon *et al*., 1999; Sims & Gamon, 2003) and the Normalized Difference Infrared Index (Fig. S2), another common method of estimating vegetation canopy water content in crop and grassland ecosystems (Hardisky *et al*., 1983; Sriwongsitanon *et al*., 2015).

### Thermal data

The Jenoptik thermal camera (IR‐TCM 640; Jena, Germany) had a broadband (7.5–14 μm) design. The FOV of the lens is 30° × 23°, leading to a roughly 1.4‐m ground pixel size. The measurement accuracy of the thermal camera was ±1.5°K within the range between 0°C and 100°C. Laboratory‐based radiometric calibration was applied to the at‐sensor digital number to calculate the at‐sensor radiance ($L_{\mathrm{at}-\text{sensor}}$). Georectification of thermal images were conducted using inflight position and posture information recorded by an independent GPS/IMU unit (NovAtel SPAN‐CPT, Calgary, Alberta, Canada).

To derive the surface temperature from the airborne thermal image, we needed to solve two major issues: (1) compensating for the atmospheric effects and (2) separating emissivity and temperature. Theoretically, the relationship between at‐sensor radiance, surface temperature and emissivity can be described as:

(Eqn 1) $$L_{\mathrm{at}-\text{sensor},\lambda }=\left[\varepsilon_{\lambda }B_{\lambda }\left(T_{s}\right)+\left(1-\varepsilon_{\lambda }\right)L_{\mathrm{atm},\lambda }^{↓}\right]\tau_{\lambda }+L_{\mathrm{atm},\lambda }^{↑}$$

where $L_{\mathrm{at}-\text{sensor},\lambda }$ is the radiance at wavelength λ measured at the sensor. $B_{\lambda }\left(T_{s}\right)$ is the Planck radiation function at temperature *T*(K) and wavelength (λ). $\varepsilon_{\lambda }$ is the emissivity of the target. $\tau_{\lambda }$ is the atmospheric transmittance. $L_{\mathrm{atm},\lambda }^{↓}$ and $L_{\mathrm{atm},\lambda }^{↑}$ are the downward and upward atmosphere radiation, respectively.

We had one broad thermal band; thus, we could only use the single‐channel method for atmospheric correction and estimation of emissivity and surface temperature (Dash *et al*., 2002; Li *et al*., 2013; Neinavaz *et al*., 2021). For the single‐channel method, a common way to apply atmospheric correction to thermal image is to rely on the radiative transfer models, such as MODTRAN. This strategy requires accurate estimation of air temperature, pressure and water vapor along the horizonal and vertical profiles of the atmosphere, which was lacking in this study. Instead, we adapted an atmospheric correction method by including temperature measurements of the three calibration tarps using a handheld infrared thermometer (IRT) (ThermoWorks, UT, USA) and thermocouple (Type T, Omega, CT, USA) linked to a datalogger (LI‐1000; LI‐COR, Lincoln, NE, USA) on the ground. According to the Stefan–Boltzmann law, the emissivity of the three calibration tarps were calculated as (*T*_IRT_/*T*_Thermocouple_)^4^. Mathematically, we can use three sets of ground measured emissivity and surface temperature of those calibration tarps to build three equations to directly solve the atmospheric variables ($\tau_{\lambda },L_{\mathrm{atm},\lambda }^{↓}$ and $L_{\mathrm{atm},\lambda }^{↑}$) in Eqn 1. We then applied the atmospheric variables to the entire thermal image to obtain the ground‐level brightness temperature.

To calculate surface temperature from ground‐level brightness temperature, a simplified NDVI^THM^ method (Sobrino *et al*., 2008) was used to estimate per‐pixel emissivity as:

(Eqn 2) $$\varepsilon =\left\{\begin{array}{c} \;\varepsilon_{s},\;{\text{NDVI}}_{s}>\text{NDVI}\\ \varepsilon_{s}+\left(\varepsilon_{v}-\varepsilon_{s}\right)\times P_{v},\;{\text{NDVI}}_{v}>\text{NDVI}>{\text{NDVI}}_{s}\\ \;\varepsilon_{v},\;\;\text{NDVI}>{\text{NDVI}}_{v} \end{array}\;\right.$$

where $\varepsilon_{s}$ and $\varepsilon_{v}$ represented the emissivity of bare soil and dense vegetation, respectively. *P*_v_ denoted the percentage of vegetation coverage, which was calculated as:

(Eqn 3) $$P_{v}=\frac{\text{NDVI}-{\text{NDVI}}_{s}}{{\text{NDVI}}_{v}-{\text{NDVI}}_{s}}$$

This method maintained the continuity at NDVI = NDVIs (*P*_v_ = 0) and NDVI = NDVI_v_ (*P*_v_ = 1). In this study, we used empirical emissivity values for dry sandy soil ($\varepsilon_{s}$= 0.95; van Wijk, 1963) and vegetation ($\varepsilon_{v}$ = 0.985; Sobrino *et al*., 2008). NDVI products were calculated using the hyperspectral image (details discussed above). Because the spatial resolution of the hyperspectral images (1 m) was finer than the thermal data (1.4 m), for each pixel in the thermal image, the NDVI value of the pixel in the hyperspectral data with shortest distance was used.

### Statistical analyses

To test the effects of management and seeding practices on relationships between field‐sampled biodiversity and remote sensing‐derived productivity (NDVI) and energy balance (albedo, WBI and surface temperature) indices, we fit three sets of linear regression models by: (1) combining all the data; (2) grouping data by management (burning and haying) practices; and (3) grouping data by seeding practices (low‐, mid‐ and high‐diversity levels). We applied analysis of covariance (ANCOVA) to test whether management and seeding practices affect the slopes and intercepts of fitted relationships. Linear regressions and ANCOVA were conducted using MATLAB (2021).

We used a nonmetric multidimensional scaling (NMDS) ordination to test the effects of management and seeding practices on plant community composition. We averaged data across 15 subplots within each plot to generate a plot‐level vegetation species percent cover dataset. We then calculated the relative cover of each plant species as its percent of total vegetation cover within each plot, excluding bare ground and litter. Species with < 5% coverage from all the plots were removed (Alday *et al*., 2013; Porensky *et al*., 2018), reducing the dataset from 157 species to 53 species. To determine the number of necessary dimensions to create an NMDS ordination (function metaMDS), we used a stress value < 0.15 based on Bray–Curtis distances (Porensky *et al*., 2018). The NMDS analysis was conducted using the vegan library (Oksanen *et al*., 2025) in R (R Core Team, 2015).

## Results

**Fig. 2:**
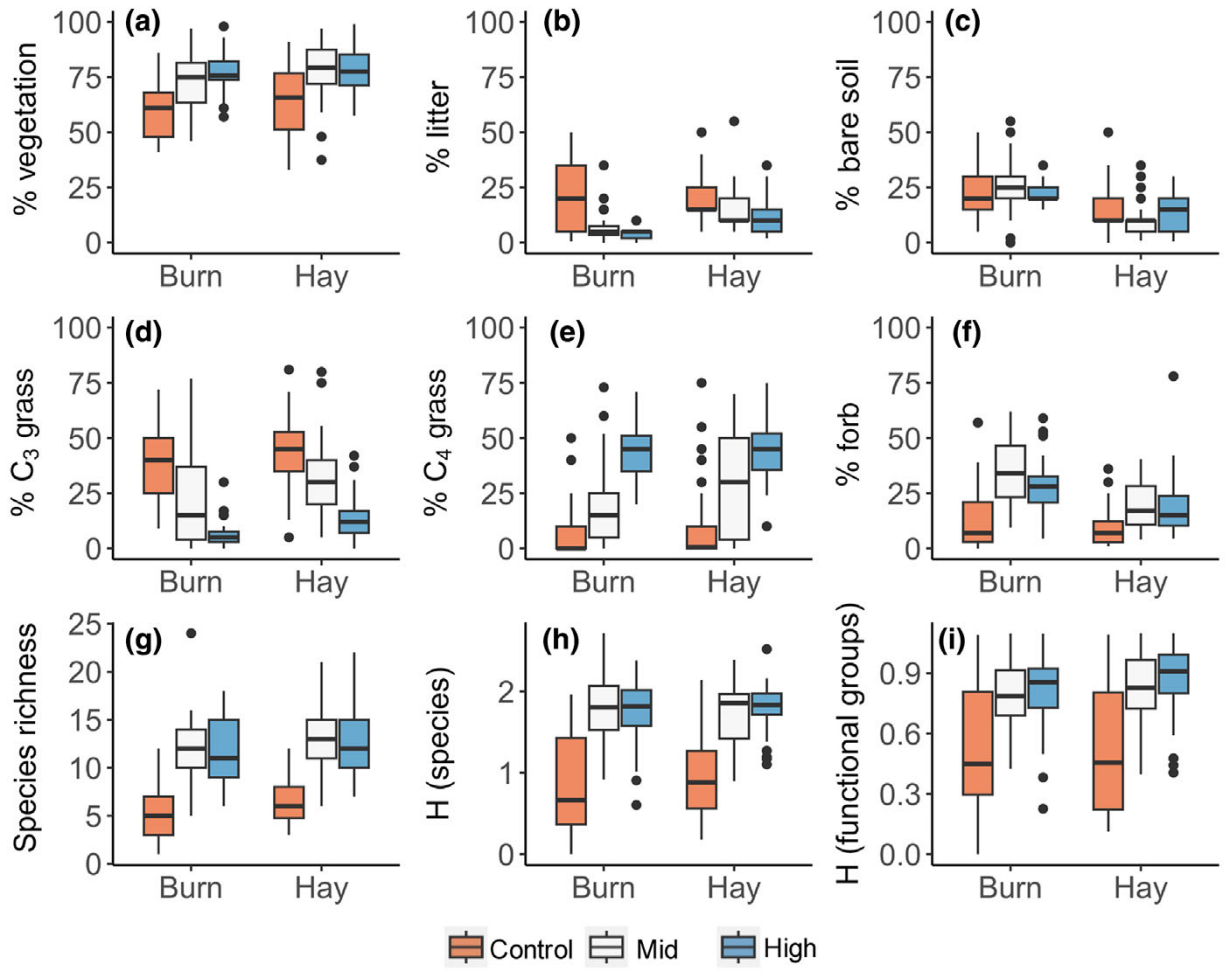
Effects of seeding treatments (control, mid‐ and high‐diversity) and managements (burning and haying) on percent cover of vegetation (a), litter (b) and bare soil (c), and three functional groups, including C_3_ grasses (d), C_4_ grasses (e) and forbs (f), species richness per square meter (g), Shannon's index based on species (h) and Shannon's index based on functional groups (i). The horizontal line within each box represents the median. The lower and upper hinges correspond to the first (25^th^ percentile) and third (75^th^ percentile) quartiles, respectively. The whiskers extend to the most extreme data points within 1.5 times the interquartile range from the lower and upper quartiles. Points beyond the whiskers are plotted individually as outliers.

Consistent effects of seeding and management treatments (burning and haying) emerged across multiple indicators of prairie productivity and energy balance from the grassland restoration experiment. Seeding and management treatments affected the percent cover of plants, litter and bare soil, as well as vegetation diversity (species and functional type composition) (Fig. 2). In general, the mid‐ and high‐diversity plots had a higher vegetation cover (Fig. 2a) and lower litter cover (Fig. 2b) than the low‐diversity control plots. More species established successfully and consistently in the mid‐ and high‐diversity plots, resulting in a lower percent cover of C_3_ grass (Fig. 2d), a higher percent cover of C_4_ grass (Fig. 2e) and forbs (Fig. 2f), and higher overall plant diversity (Fig. 2g–i) than in the control plots, in which two non‐native species, including *B. inermis* and *Convolvulus arvensis* L. dominated. Burning removed dead plant material from previous growing seasons, resulting in reduced cover of litter in the mid‐ and high‐diversity plots than hayed plots, but not in the control plots (Fig. 2b). The proportion of bare soil was also higher in the burned plots than in hayed plots (Fig. 2c). Fire slightly suppressed the proportion of C_3_ grasses (Fig. 2d) and increased the proportion of forbs (Fig. 2f), but led to no clear difference in C_4_ grass cover (Fig. 2e).

**Fig. 3:**
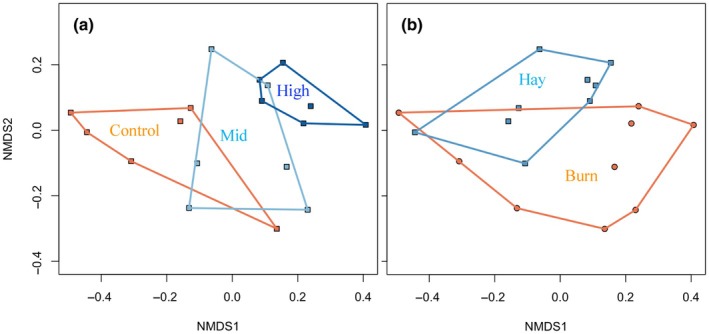
Site scores plotted on the nonmetric multidimensional scaling axes, illustrating the effects of seeding treatments (control, mid‐ and high‐diversity; a) and management (burning and haying; b) on species composition. Convex hulls were used to highlight the points clusters based on seeding and management treatments.

The NMDS ordination results further illustrated the impacts of seeding and management treatments on vegetation community composition (Fig. 3). While there were no striking distinctions in species richness (Fig. 2g) or Shannon's index (Fig. 2h,i) between the mid‐ and high‐diversity seeding treatments, plots were clustered in multivariate space, indicating different community compositions among seeding (Fig. 3a) and management (Fig. 3b) treatments. In particular, the high‐diversity plots had lower proportions of C_3_ plants (Fig. 2d) and higher proportions of C_4_ plants (Fig. 2e), leading to a higher functional diversity (Fig. 2i). Compared with the mid‐diversity mix, the higher dominance of C_4_ within the high‐diversity seed mix was apparent within burned plots. The mid‐diversity mix had some short and mid‐stature native C_4_ and C_3_ grasses, but the tall, highly competitive C_4_ grasses (Indiangrass, big bluestem and switchgrass) were excluded, which limited competition with forbs (Table S1).

**Fig. 4:**
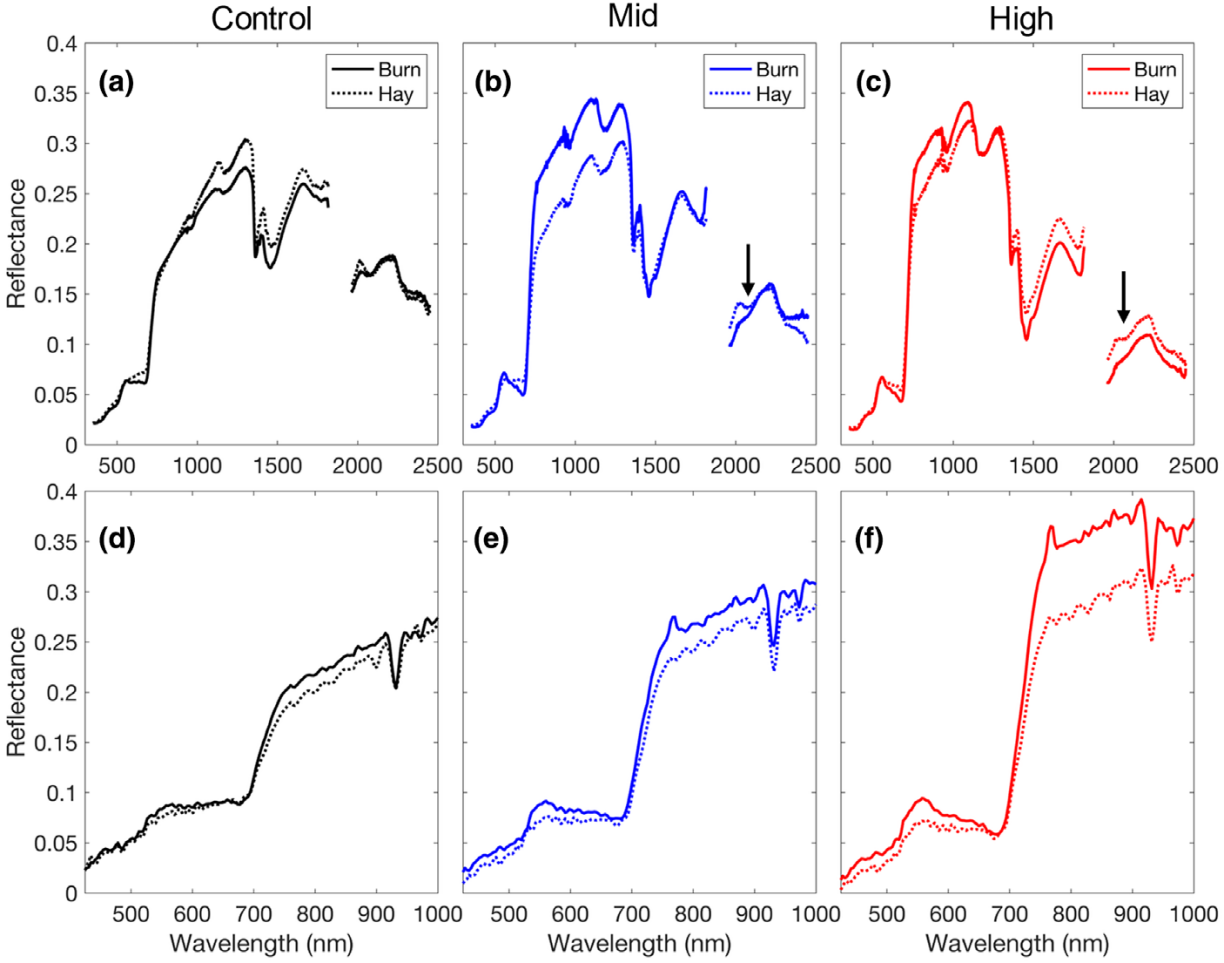
Mean reflectance spectra of transects from the six plots sampled from the ground (a, b, c) and air (d, e, f). Airborne data were extracted from the AISA Kestrel image using coordinates. The black arrow (b, c) indicates the lignin and cellulose absorption features that were missing from the burned plots. Note that the airborne data only covered the visible–near‐infrared (400–1000 nm) spectral range. The ground and airborne spectra were plotted with different spectral ranges (*X*‐axis scales).

Ground and airborne spectroscopy clearly showed the effects of seeding and management (Fig. 4). Compared with the hayed plots, the burned plots exhibited a higher surface reflectance in the VIS‐NIR spectral range (Fig. 4), although this was not clear in the ground sampling of the control plots (Fig. 4a). In the ground spectral measurements, which covered the full visible to shortwave infrared spectral range (400–2500 nm), the lignin and cellulose absorption features in the shortwave infrared were largely removed by burning (arrows; Fig. 4b,c), indicating a reduction in standing dead biomass. This was more obvious in the mid‐ (Fig. 4b) and high‐diversity (Fig. 4c) plots than in the low‐diversity control plots (Fig. 4a) due to the higher vegetation cover in these higher diversity plots. The airborne data captured the spectral difference (detected using ground measurements) among plots, but presumably with a more representative sample that measured the entire treatment plot area rather than the small subsample measured on the ground (Fig. 1). In particular, the airborne imagery recorded an increased albedo in burned plots (relative to hayed plots) and in the higher diversity plots (relative to the controls) (Fig. 4d–f). Deeper water absorption features were found in the airborne data compared with those in the *in situ* spectral data, indicating potential atmospheric correction residuals in the water bands.

**Fig. 5:**
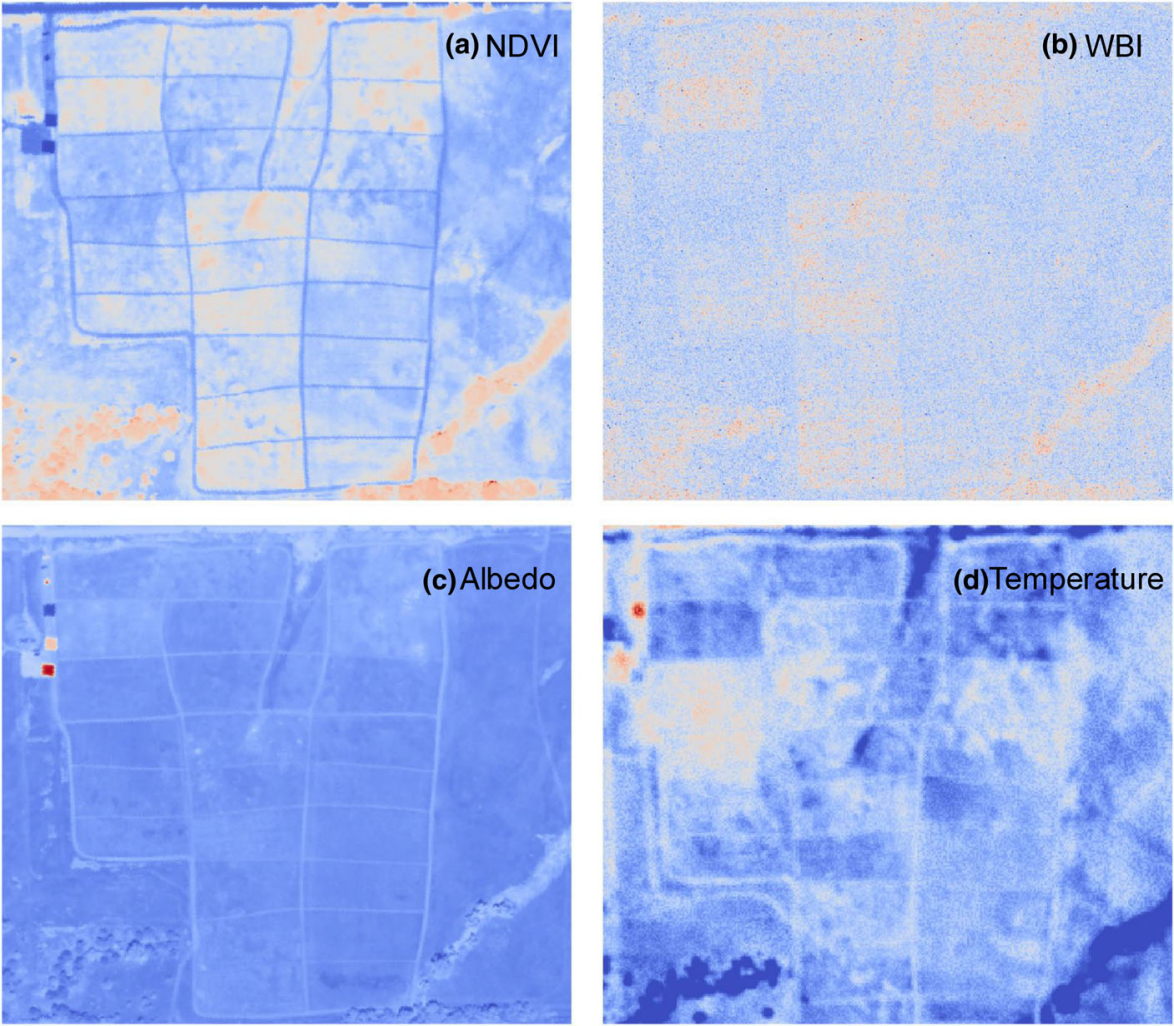
Airborne imagery revealing spatial patterns of indices related to surface energy balance, including productivity (NDVI; a), canopy water index (WBI; b), albedo (c) and surface temperature (d). For all panels, red indicates high values while blue indicates low values. The false color airborne image is shown in Fig. 1(b).

The airborne imagery revealed complex spatial patterns of remote sensing indices related to productivity (NDVI), canopy water content (WBI), albedo (integrated reflectance) and surface temperature for the contrasting restoration treatments (Fig. 5). Higher NDVI, WBI and albedo but lower temperature values (Fig. 5a–c) generally corresponded to greener vegetation and more active plant growth, which in this case was also associated with greater plant species and functional diversity. These features were associated with large observed quantities of C_4_ grasses – mostly Indiangrass (*S. nutans*) with smaller amounts of big bluestem (*Andropogon gerardii* Vitman). Conversely, areas with low NDVI, WBI and albedo (Fig. 5a–c), but high temperature (Fig. 5d), were areas dominated by C_3_ grasses, mostly *B. inermis* (smooth brome). The narrow, cool area with high NDVI roughly in the middle of the site (Plot 13, Fig. 1a) was a low, wet area of extremely thick, tall C_4_ grass growth spanning the experiment, as Plot 13 was a mid‐diversity plot that was not disked and had heavy extant C_4_ grass cover before the experiment.

**Fig. 6:**
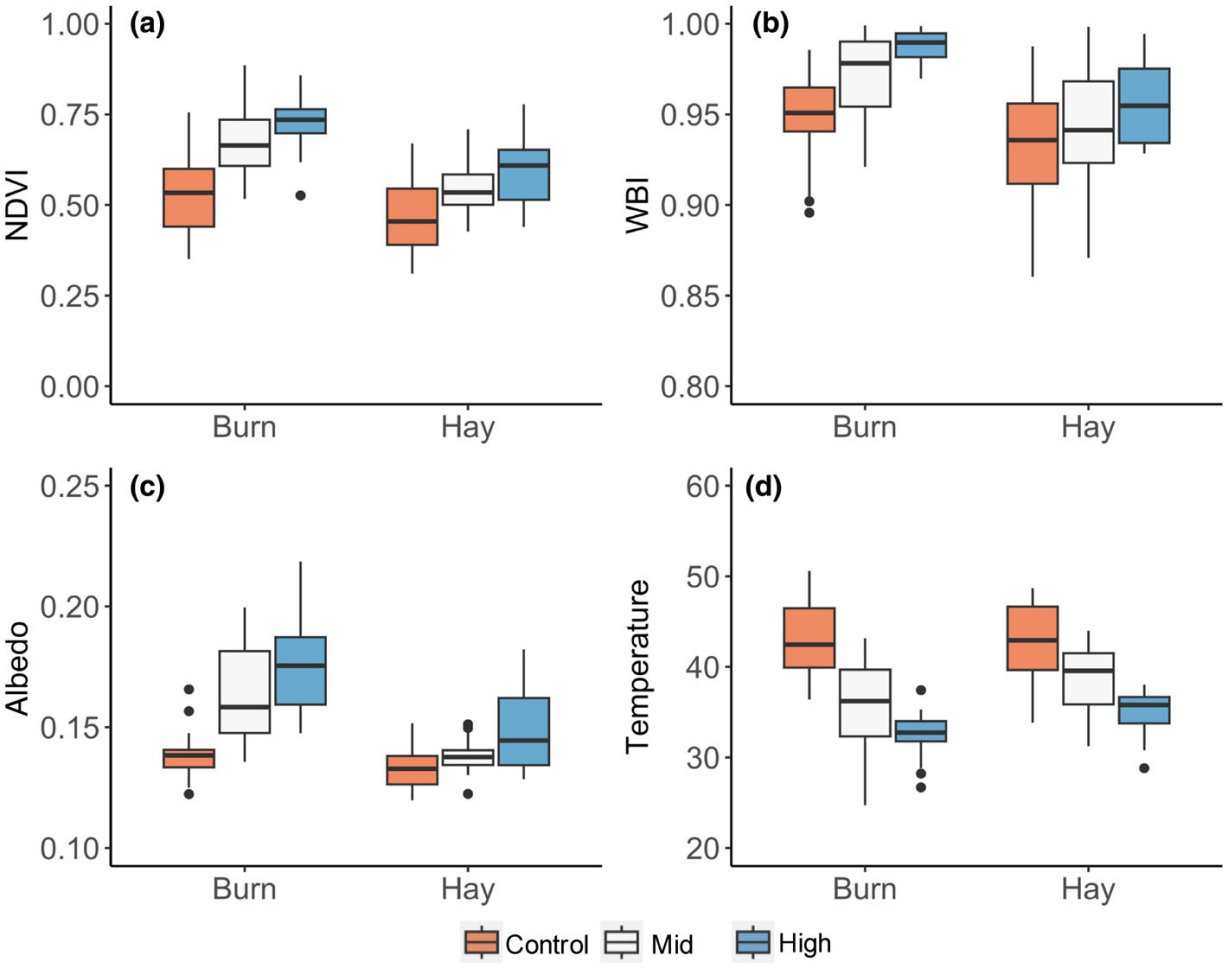
Responses of remote sensing indices related to surface energy balance, including productivity (NDVI; a), canopy water index (WBI; b), albedo (c) and temperature (d) to seeding and management (burning and haying) treatments derived from airborne imagery. The horizontal line within each box represents the median. The lower and upper hinges correspond to the first (25^th^ percentile) and third (75^th^ percentile) quartiles, respectively. The whiskers extend to the most extreme data points within 1.5 times the interquartile range from the lower and upper quartiles. Points beyond the whiskers are plotted individually as outliers.

Overall, the airborne remote sensing products illustrated the combined effects of management (burning vs haying) and seeding on productivity, vegetation water content and surface energy balance (Figs 6, S3). Burned plots had higher NDVI, WBI, and albedo and lower temperature than hayed plots, and this pattern was more obvious in the mid‐ and high‐diversity plots than in the low‐diversity control plots (Fig. 6). These findings indicate an interactive effect of seeding and management on vegetation productivity (NDVI, Fig. 6a), water content (WBI, Fig. 6b), surface reflectivity (albedo, Fig. 6c) and energy balance (temperature, Fig. 6d).

**Fig. 7:**
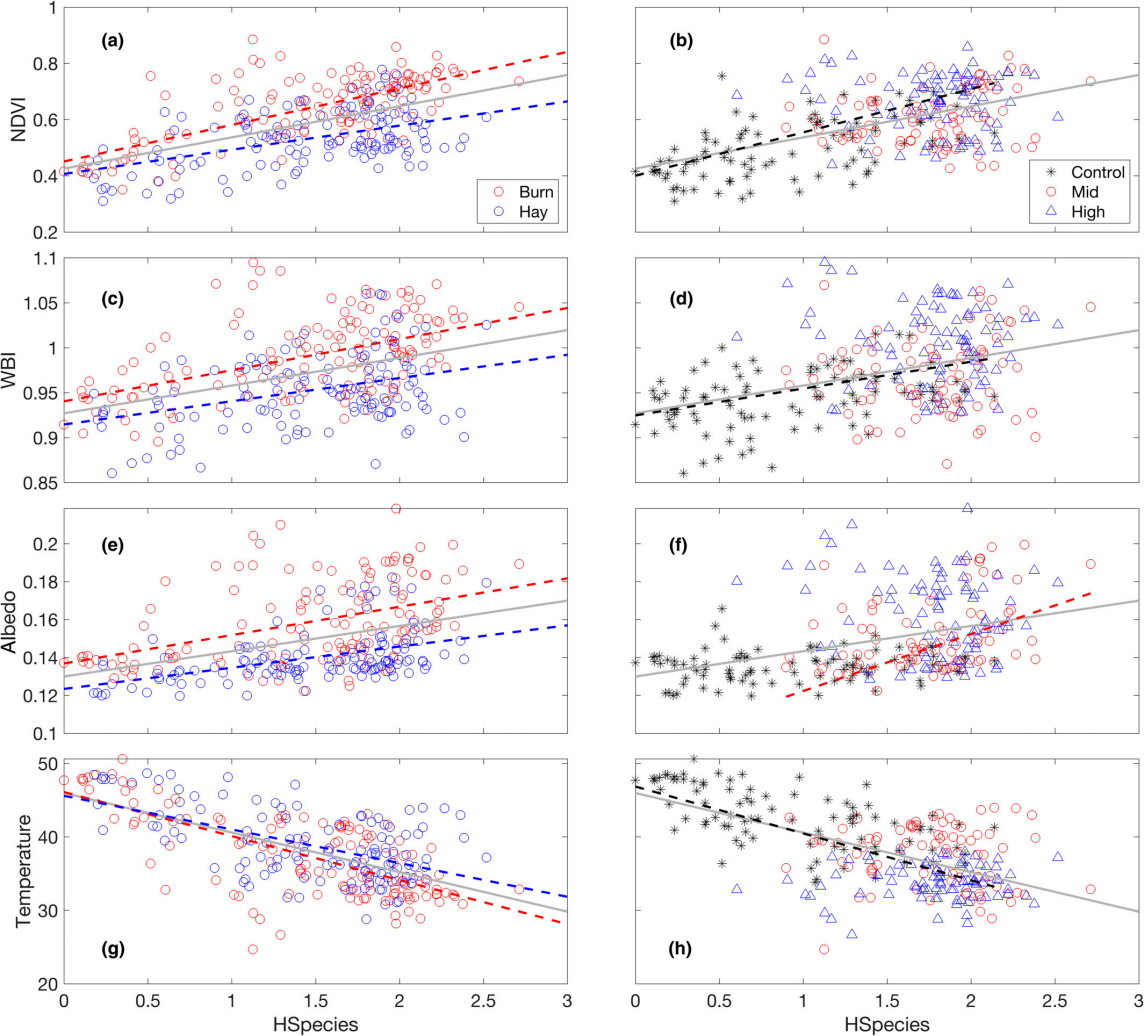
Relationships between Shannon's index based on species (HSpecies) and various remote sensing products related to surface energy balance, including productivity (NDVI; a, b), canopy water index (WBI; c, d), albedo (e, f) and temperature (g, h). Gray line indicates the relationship using all the data. Fit lines (dotted) are only shown for significant relationships (Table 3).

Strong linear relationships were found between Shannon's index based on species data (HSpecies) and vegetation indices and temperature calculated using airborne imagery (Fig. 7; Table 3). These relationships varied with management (Fig. 7a,c,e,g) and seeding (Fig. 7b,d,f,h) treatments. Burning affected the slopes of the NDVI–Shannon's index (*P* = 0.01) and the intercepts of the WBI–Shannon's index and the albedo–Shannon's index (*P* < 0.05). Neither slope nor intercept of the temperature–Shannon's index relationship was significantly different between the burned and hayed plots. When data were split by seeding treatments, there was no significant relationship between remotely sensed data and Shannon's index for the mid‐ and high‐diversity plots, except for the relationship between albedo and Shannon's index for the mid‐diversity plots (Fig. 7f). This lack of significance in the mid‐ and high‐diversity plots was because data from both hayed and burned plots were grouped together under the same diversity category when categorized by seeding treatments. For the control plots, significant relationships were found between NDVI, WBI, temperature and Shannon's index (Fig. 7b,d,h).

**Table 3:** Coefficient of determination (*r* ^2^) of relationships between Shannon's index based on species (HSpecies) and different remote sensing products.

Indices	Overall	Management		Seeding		
		Burn	Hay	Control	Mid	High
NDVI	0.297***	0.483***	0.239***	0.336***	ns	ns
WBI	0.158***	0.246***	0.13***	0.135***	ns	ns
Albedo	0.153***	0.19***	0.224***	ns	0.127**	ns
Temperature	0.39***	0.471***	0.309***	0.382***	ns	ns

Data are combined (overall) and split by management (burning vs haying) and seeding treatments (control, mid‐ and high‐diversity). Significant codes: ns, 0.05 < *P*; **, 0.001 < *P* < 0.01; and ***, *P* < 0.001. NDVI, normalized difference vegetation index; WBI, water band index.

**Fig. 8:**
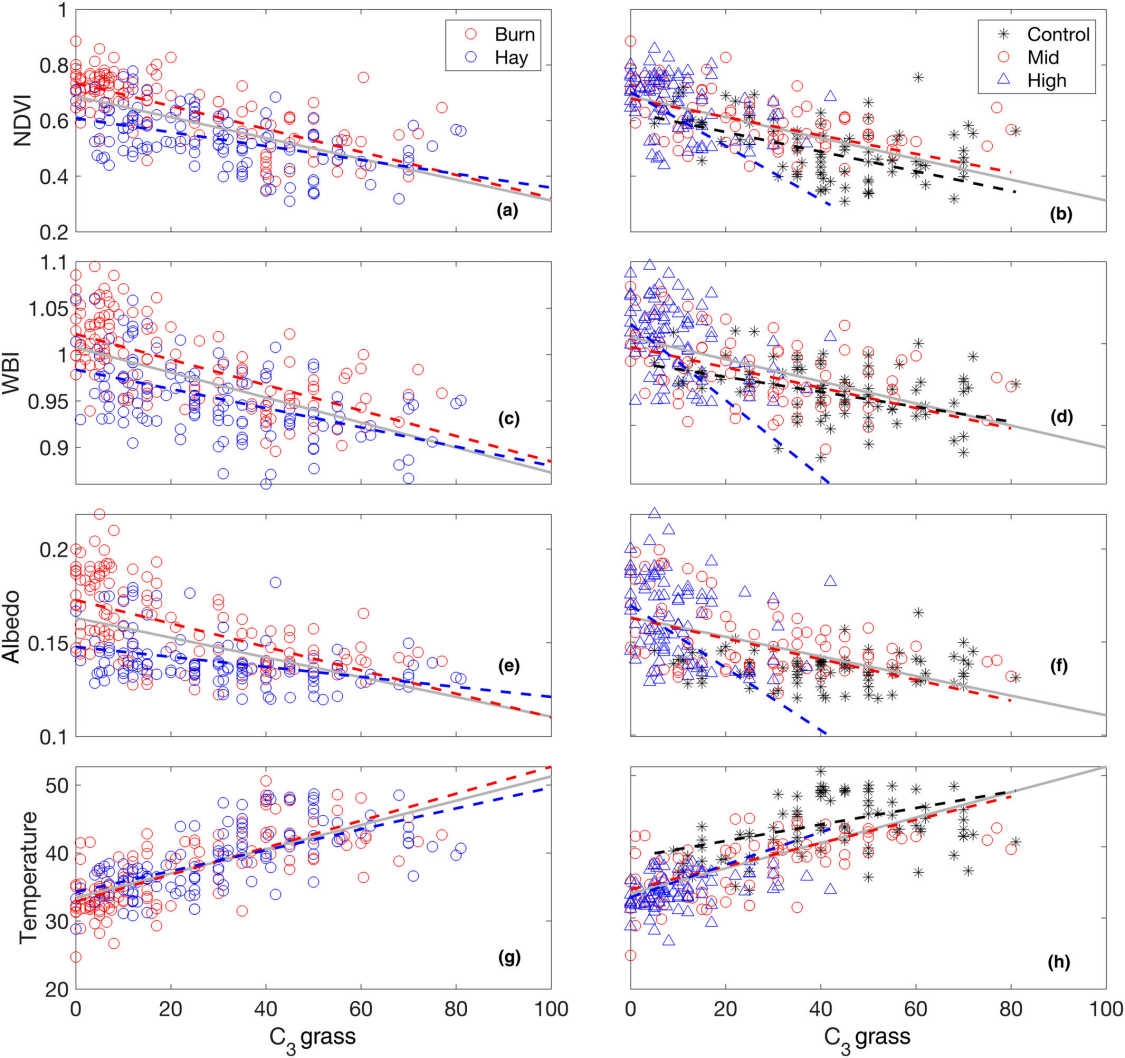
Relationships between the percent cover of C_3_ grass and various remote sensing products related to surface energy balance, including productivity (NDVI; a, b), canopy water index (WBI; c, d), albedo (e, f) and temperature (g, h). Gray line indicates the relationship using all the data. Fit lines (dotted) are only shown for significant relationships (Table 4).

Comparing the remote sensing products to the percent cover of C_3_ grasses further revealed clear effects of functional type composition on remotely sensed data (Fig. 8; Table 4). Significant differences in the intercepts, but not slopes, of productivity (NDVI)–C_3_ percent cover and canopy water content (WBI)–C_3_ percent cover were found between the burned and hayed plots. For the albedo–C_3_ percent cover relationship, both slopes and intercepts were significantly different (*P* < 0.001) between the burned and hayed plots. There was no significant difference in slopes or intercepts between the burned and hayed plots in the temperature–C_3_ percent cover relationship. For the seeding effects, the high‐diversity plots were different from the mid‐ and low‐diversity plots in the productivity–, canopy water content– and albedo–C_3_ percent cover relationships, which were presumably caused by the relatively low percent cover of C_3_ grasses in the high‐diversity plots. However, no difference was noticed in the temperature–C_3_ percent cover relationships among the seeding treatments.

**Table 4:** Coefficient of determination (*r* ^2^) of relationships between percentage cover of C_3_ grasses and different remote sensing products.

Indices	Overall	Management		Seeding		
		Burn	Hay	Control	Mid	High
NDVI	0.394	0.516	0.245	0.198	0.27	0.128
WBI	0.352	0.41	0.254	0.142	0.207	0.162
Albedo	0.279	0.35	0.155	ns	0.208	0.08
Temperature	0.5	0.546	0.422	0.142	0.37	0.127

Data are combined (overall) and split by management (burning vs haying) and seeding treatments (control, mid‐ and high‐diversity). A relationship with *P* value > 0.05 is shown as nonsignificant. NDVI, normalized difference vegetation index; WBI, water band index.

## Discussion

### Links between diversity, productivity and energy balance

In grasslands, the relationship between biodiversity, ecosystem function and energy balance is complex and multifaceted, but key findings have emerged, suggesting functional relationships that can be influenced by management choices. Our concern here is with lightly managed grasslands lacking substantial irrigation and fertilization, which characterize much of the world's rangelands. In general, higher diversity grasslands tend to have higher productivity, expressed as higher vegetation percent cover and density, and more complex leaf traits and 3D canopy structure, which can together lower the surface temperature by affecting both energy absorption, reflection, evapotranspiration and sensible heat flux (Vojtech *et al*., 2008; Milcu *et al*., 2016; Guimarães‐Steinicke *et al*., 2021). In this study, the high vegetation cover in the high‐diversity plots likely had higher thermal mass and evapotranspiration (suggested by the higher canopy water content), lowering the surface temperature (Figs 4, 5). Additionally, the higher albedo of the higher diversity plots likely contributed to a greater cooling effect and lower surface temperature.

We note that some studies have reported high productivity in less diverse grasslands (e.g. monocultures), especially when abundant water and nutrients were provided, akin to agricultural management practices (Adler *et al*., 2009; Johnson *et al*., 2010; Griffith *et al*., 2011; Brun *et al*., 2019). Thus, the diversity–productivity relationship observed here may not always occur in heavily managed systems, which often receive large resource inputs in the form of fertilizer or irrigation. These inputs, while beneficial for productivity, can lead to many adverse impacts on downstream water quality (Gamon, 2023). The loss of biodiversity can also lead to the loss of ecosystem functions both within and across trophic levels, and the effects of biodiversity loss likely get amplified at larger spatial and temporal scales (Isbell *et al*., 2011, 2018; Cardinale *et al*., 2012; Reich *et al*., 2012).

The composition of species or functional groups rather than species richness *per se* affects the energy balance in grasslands (Figs 2, 3), aligning with findings from previous studies (Petersen & Isselstein, 2015; Schouten, 2022). Under high soil moisture conditions, C_3_ grass areas would likely be cooler than C_4_ grass areas because C_3_ grasses have higher stomatal conductance and transpiration for the same amount of leaf productivity, lowering surface temperature. However, this situation likely changes with seasonal conditions: the low temperature values in our high‐diversity plots directly corresponded to the high dominance of tall C_4_ grasses, especially when burned (Fig. 2). This is probably because our data were collected in the mid‐growing season when the cool season grasses were seasonally inactive before greening up again in the fall, whereas the warm‐season C_4_ grasses retained moisture and remained active. Also, due to the dry and hot summer in our field site, little soil moisture was available for the shallow‐rooted smooth brome grass (*B. inermis*); presumably, the stomatal conductance and transpiration of this species dominating the low‐diversity plots had largely shut down at the time when we took measurements. By contrast, the deeper rooted native C_4_ grasses such as big bluestem (*A. gerardii*) remained green and were able to maintain their higher stomatal conductance and transpiration, which led to evaporative cooling of the vegetation surface. These observations suggest that the direction and magnitude of the temperature effect may differ under different conditions or times of the year.

### Fire/hay treatment modulates these effects

Management regimes (burning and haying) affect grassland diversity and modulate the coupling between vegetation and the atmosphere in complex ways (Gordijn & O'Connor, 2021). Phylogenetic lineage and lifecycle rather than photosynthetic type may determine the response of different species to fire, and this response is further affected by the frequency and timing of fire (Ripley *et al*., 2015) and fire age (time since previous fire) (Porensky *et al*., 2018). In this study, the prescribed burn occurred when the smooth brome (*B. inermis*) was fully grown but had not yet set seed and thus may have temporarily exhausted the plant's resources for further growth that year. Also, increased soil temperature and evapotranspiration associated with the postburn conditions would also have aggravated smooth brome in a drought year and encouraged it to shut down transpiration mid‐growing season. By removing dead thatch from previous seasons, suppressing C_3_ grass (mostly the smooth brome) growth, the prescribed burn stimulated native C_4_ grass growth, possibly because shade‐intolerant C_4_ grasses can grow faster than C_3_ grasses in hot and open conditions and benefit from biomass removal (Osborne & Beerling, 2005). These observations indicate that the timing of particular management treatments may be critical in attaining key management outcomes.

### The role of grassland restoration programs in climate mitigation

Ecological restoration is an effective solution to counter the loss of biodiversity and ecosystem services and also offers a powerful tool to mitigate climate change. Through energy balance (albedo and evapotranspiration), carbon uptake and storage, and other processes, terrestrial ecosystems, including grasslands, can amplify or dampen climate change rising from anthropogenic greenhouse gas emissions. Similar effects of productivity and energy balance have been noted for forests (e.g. Bonan, 2008) but have generally not been considered for grasslands, which cover roughly 40 percent of terrestrial surfaces and dominate the interannual variability in the terrestrial carbon sink (Poulter *et al*., 2014; Ahlström *et al*., 2015; Wang *et al*., 2022), offering significant climate mitigation potential. In our study, these effects were detectable in the altered productivity, albedo and surface temperature in treatment plots (Figs 2, 5, 6). While we detected enhanced productivity in more diverse plots, we did not test belowground carbon directly, but numerous studies from experimental grasslands suggest that enhanced diversity and aboveground productivity can, over time, enhance soil carbon storage (Steinbeiss *et al*., 2008; Lange *et al*., 2015). We expect that restoring diverse productive grasslands can reduce atmospheric CO_2_ content by storing carbon biomass, particularly belowground biomass. However, while restoration activities may start the recovery process relatively quickly, a full recovery of a former community and the belowground structure of a secondary grassland may take longer (Weisser *et al*., 2017; Nerlekar & Veldman, 2020).

### Conclusions and recommendations for future work

Through a multiyear grassland restoration experiment in a tallgrass prairie, we revealed a coherent response of multiple airborne remote sensing indices related to grassland biodiversity, productivity and energy balance to management and seeding strategies. Our results suggest that restoration choices can also influence feedbacks to the atmosphere and climate, both through enhanced carbon uptake (higher NDVI indicating greater plant growth) and altered energy balance (lower surface temperature and higher albedo) in certain management treatments. However, we note that more work is needed to further test the effects of grassland management treatments at various spatial and temporal scales and spanning multiple dimensions of diversity, and with varying resource inputs. In this study, we focused field sampling on vegetation community composition. In the future, including plant functional trait‐based community features (i.e. trait composition and diversity) could provide further insights into how different plant communities interact with environmental conditions and contribute to ecosystem functions and respond to environmental changes (Galán Díaz *et al*., 2022; Sperandii *et al*., 2025).

Unlike the emphasis on restoring forests to increase carbon sequestration while enhancing woody plant diversity (Gilroy *et al*., 2014), less attention has been paid to grassland restoration (Buisson *et al*., 2022). Poorly planned forest restoration efforts may establish forests in natural grasslands, undermining grassland protection and restoration (Dudley *et al*., 2020). For example, the carbon benefits of woody plants may come with accruing costs of biodiversity loss in the original grassland ecosystem, threatening the ecosystem services provided by grasslands (Veldman *et al*., 2015; Pellegrini *et al*., 2016). Although clearing forests releases carbon stored in trees, it alters the albedo and evapotranspiration patterns, leaving the overall net climate cooling or warming unsettled (Bonan, 2008; Luyssaert *et al*., 2018; Temperton *et al*., 2019). Thus, we recommend long‐term strategic investigation into the impact of grassland restoration practices, considering not only diversity and productivity but also surface–atmosphere feedbacks in the context of various management treatments that include grazing and burning, which have been an integral part of historical prairie management. Such nature‐based solutions may offer new strategies to reduce biodiversity loss, and mitigate climate change, while maintaining the productivity of prairie ecosystems.

## Competing interests

None declared.

## Author contributions

RW, JAG, and DAW designed the experiments. RW, JAG, KFEH and DAW performed the experiments and analyzed the data. RW, JAG and KFEH wrote the original draft with input from DAW and PRK.

## Disclaimer

The New Phytologist Foundation remains neutral with regard to jurisdictional claims in maps and in any institutional affiliations.

## Supporting information


**Fig. S1** Relationship between NDVI and the fraction of absorbed radiation.
**Fig. S2** Strong linear relationship between Water Band Index and Normalized Difference Infrared Index, both derived using *in situ* canopy reflectance data.
**Fig. S3** Plot‐level data showing responses of airborne remote sensing indices related to surface energy balance.
**Table S1** Mean vegetation percentage cover for each plot by species.
Please note: Wiley is not responsible for the content or functionality of any Supporting Information supplied by the authors. Any queries (other than missing material) should be directed to the *New Phytologist* Central Office.

## Data Availability

The airborne imagery (https://doi.org/10.71964/111) and species data (https://doi.org/10.71964/112) are available through the University of Nebraska Libraries research data repository (SANDY).
