# Supplementary material for: Prairie management practices influence biodiversity, productivity and surface–atmosphere feedbacks
Source: New Phytol. 2025 May 14;247(2):562–76. doi: 10.1111/nph.70195 (PMC12177289; doi:10.1111/nph.70195)
Supplement: Supplementary file 1 — Fig. S1 Relationship between NDVI and the fraction of absorbed radiation. Fig. S2 Strong linear relationship between Water Band Index and Normalized Difference Infrared Index, both derived using in situ canopy reflectance data. Fig. S3 Plot‐level data showing responses of airborne remote sensing indices related to surface energy balance. Table S1 Mean vegetation percentage cover for each plot by species. Please note: Wiley is not responsible for the content or functionality of any Supporting Information supplied by the authors. Any queries (other than missing material) should be directed to the New Phytologist Central Office. [file NPH-247-562-s001.pdf]

New Phytologist Supporting Information

Article title: Prairie management practices influence biodiversity, productivity and surface-atmosphere feedbacks

Authors: Ran Wang[1], John A. Gamon[1], Katharine F. E. Hogan[1,2], P. Roxanne Kellar[3], David A. Wedin[1]

[1] School of Natural Resources, University of Nebraska-Lincoln, Lincoln, Nebraska 68583, USA

[2] Lauritzen Gardens, Omaha, Nebraska 68108, USA

[3] Department of Biology, University of Nebraska Omaha, Omaha, Nebraska 68182, USA

Article acceptance data: 15 April 2025

We calibrated NDVI against the fraction of absorbed radiation by standing biomass (F_{APAR}), a primary driver of carbon uptake in grasslands and croplands, and also an indicator of green leaf area index and biomass accumulation (Fig. S1). Prairie data were collected from Bobcat Prairie (July 20-22, 2022) and soybean and maize crops data were collected from the Eastern Nebraska Research, Extension and Education Center near Mead Nebraska (July 18, 2018). F_{APAR} was calculated using above and below-canopy light intensity measured using a line quantum sensor (ACCUPAR LP-80, Meter, Pullman WA, USA).

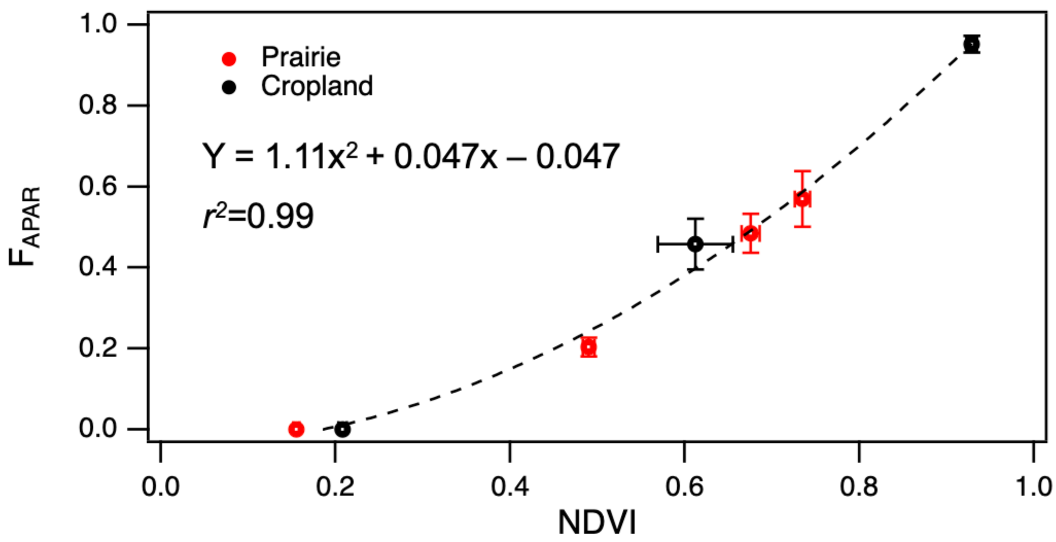

Fig. S1. Relationship between NDVI and the fraction of absorbed radiation (FAPAR). A second order polynomial function was used to fit the FAPAR-NDVI relationship.

When tested using the in situ remote sensing data, a linear relationship was found between the water band index (WBI) and the Normalized Difference Infrared Index (NDII; (Hardisky *et al.*, 1983)), another common method of estimating vegetation canopy water content in crop and grassland ecosystems (Sriwongsitanon *et al.*, 2015). Note that in addition to NDII, the WBI has also been shown to be closely related to several other metrics of vegetation content derived from reflectance, indicating the functional equivalence of these metrics under the conditions of our study.

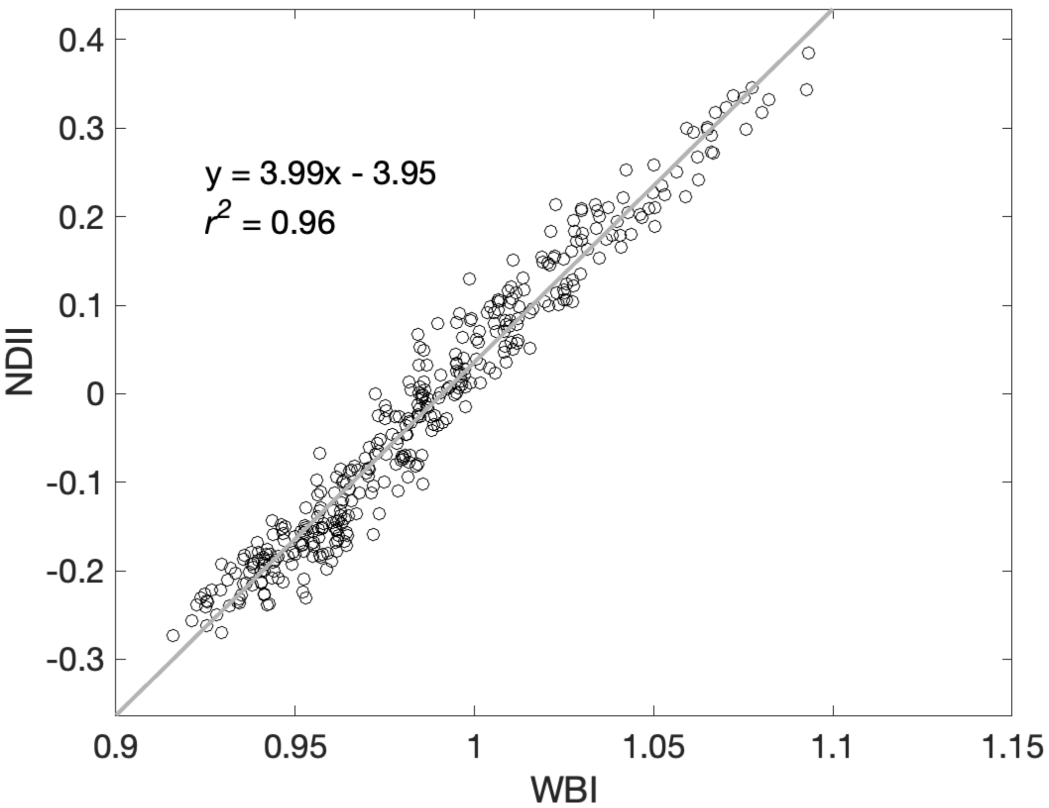

Fig. S2. Strong linear relationship between WBI and NDII, both derived using in situ canopy reflectance data.

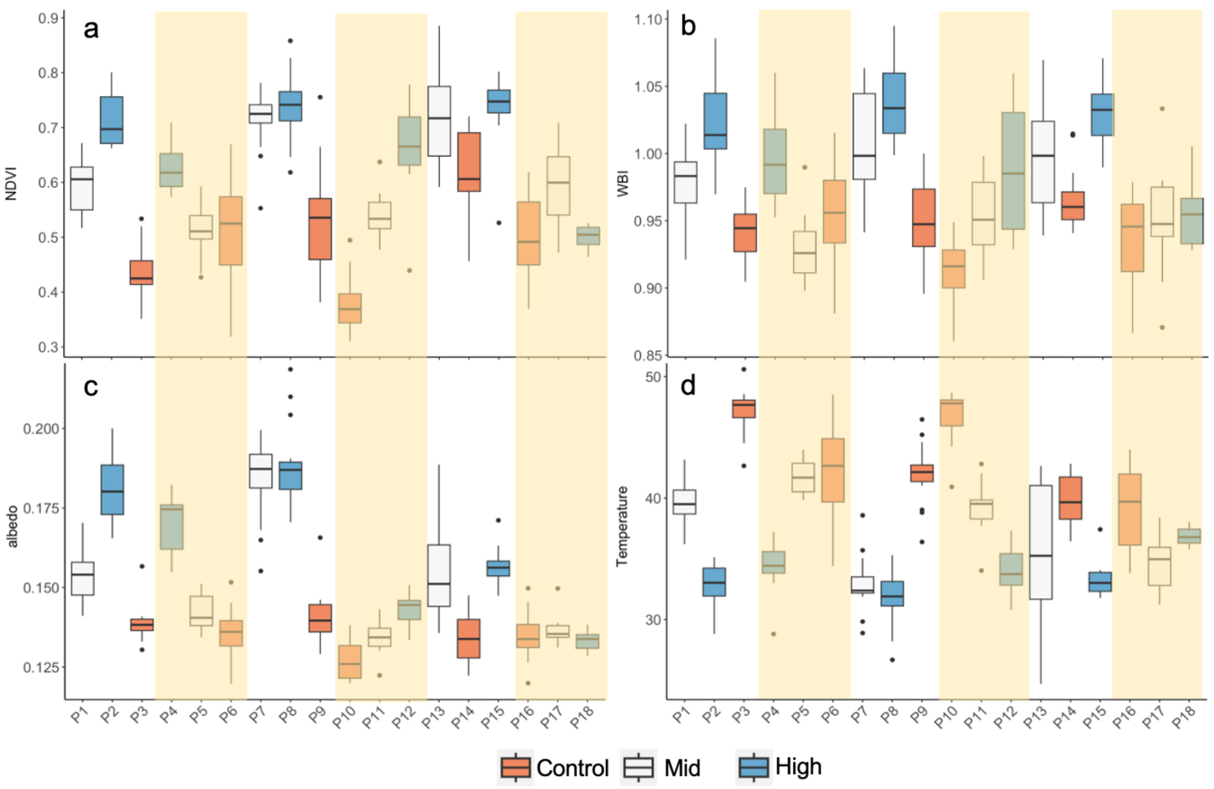

Fig. S3. Plot level data showing responses of airborne remote sensing indices related to surface energy balance, including productivity (NDVI; a), canopy water index (WBI; b), albedo (c), and temperature (d) to seeding and management (burning and haying) treatments. The yellow shading indicates blocks that received annual haying since 2019. The non-shaded plots were burned on May 10, 2022.

Table S1 Mean vegetation percentage cover for each plot by species.

Scientific Name	P1	P2	P3	P4	P5	P6	P7	P8	P9	P10	P11	P12	P13	P14	P15	P16	P17	P18
Abutilon theophrasti Medik.	0	0	0	0	0	0	0	0	0	0	0	0	0	0	0	0	0	0
Acer saccharinum L.	0.50	0	0	0	0	0	0	0	0	0	0	0	0	0	0	0	0	0
Achillea millefolium L.	0.50	0	0	0	0.75	0	1.33	0	0	0	0	0	0	0	0	0	1.00	0
Amaranthus retroflexus L.	0	0	0	0	0	0	0	0	0	0	0	0	0	0	0	0	0	0
Ambrosia artemisiifolia L.	0	0	0	0	0	0.50	0	0	0	0	0	0	0.67	0	0	0	0	0
Ambrosia psilostachya DC.	1.19	0.75	1.00	0	0	1.00	1.25	1.14	2.10	0.50	0.50	0.50	1.00	5.33	0.90	1.00	0	0.50
Ambrosia trifida L.	0	0	0	0	0	0	0	0	0	0	0	0	0	0	2.00	0	0	0
Amorpha canescens Pursh	0	1.00	0	1.00	1.00	0	0	0	0	0	0	0	0	0	0.75	0	0	0.83
Andropogon gerardii Vitman	0	20.91	0	15.20	0	0	0	34.67	0	0	0	17.20	9.71	5.00	17.00	15.67	19.20	6.14
Antennaria neglecta Greene	0.75	1.00	0	0	1.00	5.67	1.60	0	1.00	0	3.00	1.00	0	0	1.00	3.00	5.00	2.00
Apocynum cannabinum L.	0	0	0	0	0	0	0	0	0	0	0	0	0	1.50	0	0	1.00	0
Artemisia ludoviciana Nutt.	0	0	0	1.00	0	0	0.50	0	0	0	0	0	0	0	0	0	0	0
Asclepias syriaca L.	0	0	0.50	0	0	0	0	0	0	0	0	0	0	0	0	0	0	0
Asclepias tuberosa L.	0	0	0	0	0	0	0	0	0	0	0	0	0	0	0	0	0	0
Asclepias verticillata L.	0.50	0.50	0	0	0.50	0	0.50	1.00	4.00	0.50	0	0	1.00	1.00	0.50	0	0	0.50
Asclepias viridis Walter	0	0	0	0	0	0	0	0	0	0	0	0	0	0	0	0	0	0
Astragalus canadensis L.	8.00	1.00	0	2.00	1.25	0.50	0	0	0	0	4.30	0.75	5.67	5.00	5.00	0	1.80	10.00
Astragalus crassicarpus Nutt.	1.00	0	0	0	0	0	0	0	0	0	0	0	0	0	0	0	0	0
Baptisia bracteata Muhl. ex Elliott	0	0	0	0	0	0	0	0	0	0	0	0	1.00	0	0	1.00	0	0
Bouteloua curtipendula (Michx.) Torr.	3.64	1.33	0	10.00	2.80	0	1.00	8.17	0.50	0	0	2.50	5.67	0	1.75	0	14.00	3.33
Brassica nigra (L.) W.D.J.Koch	0	0	0	0	0	0	0	0	0	0	0	0	0	0	0	0	0	0
Brickellia eupatorioides (L.) Shinners	4.85	4.25	0	4.00	3.46	4.32	6.00	3.75	4.50	1.50	4.88	5.21	1.75	10.50	2.44	3.50	3.00	1.57
Bromus inermis Leyss.	30.47	7.92	45.00	12.43	26.43	35.63	6.21	4.11	50.80	38.13	25.36	9.33	9.00	11.94	1.88	43.21	12.08	6.27
Bromus japonicus Thunb. ex Murr.	0	0	0	0	0	0	0	0	0	0	0.50	0	0	0	0	0	0	0
Callirhoe alcaeoides (Michx.) A.Gray	0	0	0	0	0	0	0	1.00	0	0	0	0	0	0	0	0	0	0

Calylophus serrulatus (Nutt.) P.H.Raven	0	0	0	0	0	0	0	0	0	0	0	0	0	0	0	0	0	0
Cannabis sativa L.	0	0	0	0	0	0	0	0	0	0	0	0	0	0	0	0	0	0
Carex sp.	0	3.00	0	1.00	1.00	10.00	2.00	13.33	3.00	0	0	0.83	13.75	3.86	2.50	2.50	1.00	3.17
Ceanothus americanus L.	0	0	0	0	0	0	0	0	0	0	0	0	0	0	0	0	0	0
Chamaecrista fasciculata (Michx.) Greene	2.20	0	0	1.00	0.83	1.86	5.21	1.42	5.67	0	1.00	0	3.54	5.80	1.00	1.00	1.00	0
Chamaesyce maculata (L.) Small	0	0	0	0	0	0	0	0	0	0.50	0.50	0	0	0	0.50	0	0	0
Cirsium altissimum (L.) Spreng.	0	0	0	1.00	0	0	0.75	0	0	0	0	0.50	0.75	0	0	1.00	0	0
Cirsium arvense (L.) Scop.	0	0	0	0	0	0	0	0	0	0	0	0	0	0	0	0	0	0
Cirsium flodmanii (Rydb.) Arthur	0	0	0	0	0	0	0	0	0	0	0	0	0	0	0	0	0	0
Convolvulus arvensis L.	3.17	1.67	3.12	0	0	1.00	1.00	0	1.25	0.67	0.75	0.67	0	0	0	1.00	1.00	0
Conyza canadensis (L.) Cronquist	0	0	0	0	0	0	0	0	0	0	0	0	0	0	0	0	0	0
Cornus drummondii C.A.Mey.	0	0	0	1.00	0	0	3.00	1.50	0	0	0	0	0	0	2.00	1.00	0	1.00
Crotalaria sagittalis L.	0	0	0	0	5.00	0.75	2.00	0	0	0	0.50	30.00	7.67	1.50	1.50	0	0	1.93
Cynanchum laeve (Michx.) Pers.	0	0	0	0	0	0	0	0	0	0	0	0	0	0	0	0	0	0
Dactylis glomerata L.	0	0	0	0	0	0	0	0	0	0	0	0	0	0	0	0	0	0
Dalea candida Michx. ex Willd.	1.50	1.00	0	1.00	2.00	0	2.40	1.00	0	0	1.21	1.00	2.33	0	0	0	1.20	1.00
Dalea leporina (Aiton) Bullock	0	0	0	0	0	0	0.50	0	0	0	0	0	0	0	0	0	0	0
Dalea purpurea Vent.	1.00	0	0	5.25	3.40	0	1.50	0	0	0	2.88	0	4.00	0	1.00	0	1.00	0
Delphinium carolinianum Walter	0	0	0	0	0	0	0	0	0	0	0	0	0	0	0	0	0	0
Desmanthus illinoensis (Michx.) MacMill. ex B.L.Rob. & Fernald	0	0.50	0	0	0.75	0	0	2.00	0	0	1.00	3.50	5.00	0	0	0	0	0
Desmodium canadense (L.) DC.	3.67	7.00	0	3.25	0.50	0	2.25	6.20	0	0	0.50	0	1.00	0	11.43	1.00	0	1.00
Desmodium illinoense A.Gray	0.50	4.17	0	0	1.00	1.00	2.33	3.00	0	0	1.00	0	1.33	16.00	2.00	0	1.00	0
Dianthus armeria L.	0	1.00	0	1.35	2.33	2.07	0	0	0	1.59	1.75	1.00	0.50	0	0.50	1.73	1.00	0.63
Dichanthelium acuminatum (Sw.) Gould & C.A.Clark	0	0.50	0	0	1.00	2.25	1.33	5.00	3.00	0	0	0	1.00	2.00	1.43	2.00	4.25	2.38
Dichanthelium oligosanthes (Schult.) Gould	0	0	0	0	1.50	0.50	5.67	0	0	0	5.00	0.83	7.88	3.60	1.83	1.50	0	1.50
Digitaria ischaemum (Schreb.) Schreb. ex Muhl.	0	0	0	0	0.50	0	0	0	0	0	0	1.00	1.00	0	0	0	0	0
Echinochloa crus-galli (L.) P.Beauv.	0	0	0	0	0	0	0	0	0	0	0	0	0	0	0	0	0	0

Elymus canadensis L.	0	0	0	0	0	0	0	0	0	0	0	0	0	0	1.00	0	0	0.83
Eragrostis capillaris (L.) Nees	0	0	0	0	0	0	0	0	0	0	0	0	0	0	0	0	0	0
Eragrostis spectabilis (Pursh) Steud.	1.00	0	0	1.00	2.80	1.00	0	0	0	0	5.25	1.00	0	0	5.00	0	0	2.00
Erechtites hieraciifolius (L.) Raf. ex DC.	0	0	0	0	0	0	0	0	0	0	0	0	0	0	0	0	0	0
Erigeron annuus (L.) Pers.	0	0	0	0	0	0	0	0	0	0	0	0	0	0	0	0	0	0
Erigeron strigosus Muhl. ex Willd.	0	0	0	0	0	0	0	0	0	0	0	0	0	0	0	0	0	0.75
Eupatorium altissimum L.	0	0	0	0	0	0	0	0	0	0	0	0	0	0	0	0	0	0
Euphorbia nutans Lag.	0	0	0	0	0	0	1.10	0	1.00	0	0	0	0	0	0	0	0	0
Euthamia graminifolia (L.) Nutt.	0	0	0	0	0	0	1.00	0	0	0	0	0	0	0	0	0	0	0
Galium aparine L.	0	0	0	0	0	0	0	0	0	0	0	0	0	0	0	0	0	0
Gleditsia triacanthos L.	0	0	0	0	0	0	0	0	0	0	0	0	0	0	0	0	0	0
Grindelia squarrosa (Pursh) Dunal	0	0	0	0	0	0	0	0	0	0	0	0	0	0	0	0	0	0
Hedeoma hispida Pursh	0	0	0	0	0	0	0	0	0	0	0	0	0	0	0	0	0	0
Helianthus annuus L.	0	0	0	0	0	0	0	0	0	0	0	0.50	0	0	0	0	0	0
Helianthus grosseserratus M.Martens	0	0	0	0	0	0	5.00	15.00	0	0	0	0	0	0	2.00	0	2.00	0
Helianthus maximiliani Schrad.	0.75	10.53	5.00	4.09	0	0	0	9.31	0	0	0	3.55	0	0	9.00	0	0	2.50
Helianthus pauciflorus Nutt.	0	1.33	0	1.00	0	0	1.00	0	0	0	10.00	0	2.50	0	0.50	0	0	1.00
Helianthus petiolaris Nutt.	0	0	0	0	0	0	0	0	0	0	0	0	0	0	0	0	0	0
Helianthus tuberosus L.	0	0	0	0	0	0	0	0	0	0	0	0	0	0	0	0	0	0
Heliopsis helianthoides (L.) Sweet	0	0	0	0	0	0	5.00	0	0	0	0	0	0	0	0	0	0	0
Hieracium longipilum Torr.	2.25	0	0	1.00	1.00	0	1.25	0	0	0	0.50	0	0	0	1.00	1.00	0	1.00
Hypericum perforatum L.	0	0	0	0	0.50	0	0	0	0	0	0	0	0	0	0	0	0	0
Juncus sp.	0	0	0	0	0	0	0	0	0	0	0	0	0	0	0	0	0	0.50
Juniperus virginiana L.	0	0	0	0	0	0	0	0	0	0.50	0.50	0	0	0	0	0	0	0
Koeleria macrantha (Ledeb.) Schult.	0	0	0	1.00	10.33	0	0	0	0.50	0	1.14	0	0	0	1.00	0	1.50	0
Lactuca sp.	1.00	0	0	0	0	0	0	0	0	0	1.00	0	0	0	0	0	0	0
Lathyrus pratensis L.	0	0	0	0	0	0	0	0	0	0	0	0	0	0	0	0	0	0

Lepidium densiflorum Schrad.	0	0	0	1.00	0	0	0	0	0	0	0.50	1.00	0	0	0	0	1.00	0.50
Lespedeza capitata Michx.	5.00	0	0	2.00	1.57	2.25	1.00	3.00	0	0	0.67	2.33	3.38	4.33	0	1.00	2.40	2.39
Lespedeza cuneata (Dum. Cours.) G.Don	0	0	0	0	1.50	0	4.33	8.00	0	0	0	0	0	0	0	0	3.00	0
Liatris punctata Hook.	0	0	0	0	0	0	1.00	2.00	0	0	0	0	0	0	0	0	0	0
Liatris pycnostachya Michx.	0	0	0	0	0	0	0	0	0	0	0	0	0	0	0	0	0	0
Liatris spicata (L.) Willd.	0	0.50	0	0	0	0	0	0	0	0	0	0	0	0	0	0	0	0
Linum sulcatum Riddell	0	0	0	1.00	0.50	1.67	0	0	0	0.50	0.50	0.50	0	0	0	1.00	0	0.88
Lobelia spicata Lam.	0	0	0	0	0	0	0	0	0	0	0	0	0	0	0	1.00	0	0
Lotus unifoliolatus (Hook.) Benth.	0	1.00	0	1.00	1.10	0	0	0	0	0	3.56	0.71	1.00	1.00	1.13	0	0	0.67
Medicago lupulina L.	0	0	0	0	0	0	0	0	0	0	0	0	0	0	0	0	0	0
Medicago sativa L.	0	0	0	0	0	0	0	0	0	0	0	0	0	0	0	0	0	0
Melilotus officinalis (L.) Pall.	5.00	1.00	0	7.78	3.25	12.44	0.75	1.00	2.42	0	12.83	26.0	1.00	2.71	0.50	1.00	0	0
Monarda fistulosa L.	2.64	2.72	0	1.07	1.10	0	2.78	2.33	0	0	0	0.83	1.25	0	1.00	0	1.00	0.67
Morus alba L.	0	1.00	0	0	0	0	0	0	0	0	0	0	0	0	0	0	0	0
Oenothera biennis L.	1.00	0	0	0	0	0	0	0	0	0	0	0	0	0	0	0	0	0
Oenothera curtiflora W.L.Wagner & Hoch	0	0	0	0	0	1.00	0	0	0	0	0	0	0	0	0	0	0	0
Oligoneuron rigidum (L.) Small	2.14	2.50	0	1.90	4.63	5.17	8.60	3.14	1.00	0	0.88	2.67	4.20	1.67	3.31	1.00	1.00	1.28
Oxalis dillenii Jacq.	0	0	0	0	0	0	0	0	0	0	0	0	0	0	0	0	0	0
Oxalis stricta L.	0	0	0	1.00	0	0	0	0	0	0	0	0.50	0	0	0	0	0	0
Panicum virgatum L.	5.00	11.2	0	6.00	5.00	0	0	2.00	0	0	0	0.75	10.5	40.0	1.00	0	0	0
Penstemon tubaeflorus Nutt.	0	0	0	0	0	0	0	0	0	0	0	0	0	0	0	0	0	0
Phleum pratense L.	0	0	0	0	0	0	0	0	0	0	0	0	0	0	0	0	0	0
Physalis heterophylla Nees	1.00	0	0	4.00	0	0	0	0	0	0	0	0	1.00	0	0	0	0	0
Physalis longifolia Nutt.	0	1.00	0	0	0.50	0	0	0	0	0.75	1.00	0	0	0	0	1.00	0	0
Physalis sp.	0	0	0	0	0	0	0	0	2.00	0	0	0	0	0	0	0	0	0
Plantago major L.	10.00	0	0	0	0	0	0	0	0	0	0	0	0	0	0	0	0	0
Poa pratensis L.	7.92	3.86	0	3.21	8.10	4.69	3.36	2.25	3.25	6.52	12.46	3.38	22.43	7.38	3.71	5.67	6.15	8.40

Polygonum convolvulus L.	0	0	0	0	0	0	0	0	0	0	0	0	0	0	0	0	0	0
Polygonum lapathifolium L.	0	0	0	0	0	0	0	0	0	0	0	0	0	0	0	0	0	0
Polygonum sp.	0	0	0	0	0	0	0	0	0	0	0	0	0	0	0	0	0	0
Populus deltoides W.Bartram ex Marshall	0	0.50	0	0	0	0	0	0	0	0	0	0	0	0	0	0	0	0
Potentilla arguta Pursh	0	0	0	0	0	0	0	0	0	0	0	0	0	0	0	0	0	0
Potentilla norvegica L.	0	0	0	0	0	0	0	0	0	0	0	0	0	0	0	0	0	0
Potentilla recta L.	0	0	0	0	0	0	0	0	0	0	0	0	0	0	0	0	0	0
Prunus americana Marshall	0	0	0	0	0	0	0	0	0	0	0	0	0	0	0	0	0	0
Pseudognaphalium obtusifolium (L.) Hilliard & B.L.Burtt	0	0	0	0	0	0	0	0	0	0	0	0	0	0	0	0	0	0
Ratibida columnifera (Nutt.) Wooton & Standl.	1.13	0	0	0	0	0	3.00	0	0	0	0	0	4.00	0	0	0	1.00	0
Ratibida pinnata (Vent.) Barnhart	0	0	0	0	0.50	0	1.33	0	0	0	0.50	0	0	0	0	0	0	0
Rhus glabra L.	0	0	0	0	14.0	0	0	18.75	15.0	0	0	0	0	0	0	0	0	0
Rosa arkansana Porter	0	0	0	0	0	0	0	0	0	0	0	0	0	0	0	0	0	0
Rosa woodsii Lindl.	0	0	0	0	0	0	0	0	0	0	0	0	0	0	0	0	0	0
Rudbeckia hirta L.	0.92	0	0	1.67	1.38	3.00	0	1.00	0	0	1.00	0.50	1.00	0	0	1.00	1.00	0.50
Rumex crispus L.	0	0	0	0	0	0	0	0	0	0	0	0	0	0	0	0	0	0
Salvia azurea Michx. ex Lam.	2.80	0	0	0	1.50	0	1.67	0	0	0	0	0	1.00	0	0	0	0	0
Schedonorus arundinaceus (Schreb.) Dumort.	0	0	0	1.00	0	2.00	0	0	0	0	0	0	0	0	0	0	0	0
Schizachyrium scoparium (Michx.) Nash	3.50	9.25	5.00	3.25	5.33	5.00	6.00	7.50	0.50	0	15.00	11.22	2.83	0	8.89	2.00	23.00	14.23
Securigera varia (L.) Lassen	0	0	0	0	0	0	0	0	0	0	0	0	0	0	0	0	0	0
Setaria pumila (Poir.) Roem. & Schult.	2.33	1.50	0	0	0	0	2.17	0	5.00	0	0	0	0	1.00	0	0	0	0
Silphium integrifolium Michx.	0	0	0	0	0	0	0	0	0	0	0	0	0	0	1.00	0	1.00	0
Silphium laciniatum L.	0	0	0	0	0	0	0	0	0	0	0	0	0	0	0	0	0	0
Solanum carolinense L.	1.00	0	0	0	0	0	0	0	0	0	0	0	0	0	0	0	0	0
Solidago altissima L.	0	0	0	0	0	0	0	0	0	0	0	0	0	0	0	0	0	0
Solidago canadensis L.	8.88	1.25	0	1.50	1.08	0.75	21.67	5.00	1.00	0	0	0	3.22	15.00	5.69	1.00	2.25	1.07
Solidago gigantea Aiton	0	0	0	0	0	0	0	0	0	0	0	0	0	0	0	0	0	0

Solidago missouriensis Nutt.	0	0	0	1.00	0	3.50	8.67	0	0	0	0	0.50	0	2.00	0.83	0	0	1.75
Solidago sp.	0	0	0	3.00	0	0	0	0	0	0	0	0	0	0	0	0	0	0
Solidago speciosa Nutt.	10.67	2.33	0	1.00	1.00	1.00	6.75	0	0	1.00	0.63	0.75	7.50	1.00	1.00	0	1.00	0
Sorghastrum nutans (L.) Nash	1.00	21.13	0	26.07	8.00	19.63	20.77	10.00	8.60	2.00	25.42	22.67	20.00	22.00	21.79	33.13	26.50	29.00
Sporobolus cryptandrus (Torr.) A.Gray	0	2.67	0	7.13	0	0	0	0	0	0	0	0	0	0	0	0	0	0
Sporobolus heterolepis (A.Gray) A.Gray	0	0	0	0	0	0	0	0	0	0	0	0	0	0	0	0	0	0
Sporobolus compositus (Poir.) Merr.	4.00	0	0	25.0	4.14	0	0	0	3.20	0	50.0	4.33	4.67	0	4.40	0	12.5	10.0
Symphoricarpos orbiculatus Moench	0	0	0	0	0	0	0	0	0	0	0	0	0	0	0	0	0	0
Symphyotrichum cordifolium (L.) G.L.Nesom	0	0	0	0	0	0	0	0	0	0	0	0	0	0	0	0	0	0
Symphyotrichum ericoides (L.) G.L.Nesom	3.05	3.60	1.00	1.42	2.50	1.00	5.30	2.23	5.00	0.71	1.30	4.50	2.75	4.25	1.45	1.00	1.33	1.63
Taraxacum officinale F.H.Wigg.	0	0	0	0	0	0	0	0	0	0	0	0	0	0	0	0	0	0
Thlaspi arvense L.	0	0	0	0	0	0	0	0	0	0	0	0	0	0	0	0	0	0
Tragopogon dubius Scop.	0	0	0	0	0	0	0	0	0	0	0	0	0	0	0	1.00	0	0
Tribulus terrestris L.	0	0	0	0	0	0	0	0	0	0	0	0	0	0	0	0	0	0
Trifolium pratense L.	0	0	0	0	0	0	0	0	0	0	0	0	0	0	0	5.00	0	0
Trifolium repens L.	0	0	0	0	0	0	0	0	0	0	0	0	0	0	0	0	0	0
Triodanis perfoliata (L.) Nieuwl.	0	0	0	0	0	0	0	0	0	0	0	0	0	0	0	0	0	0
Ulmus pumila L.	0	0	0	0	0	0	5.00	0	0	0	0	0	0	0	0	0	0	0
Verbena stricta Vent.	3.00	0	0	0	0	0	5.00	0	0	0	0.75	0.50	0	0	0	0	1.00	0
Vernonia baldwinii Torr.	3.00	5.50	0	0	0	0	9.50	5.00	0	0	0	1.00	0	10.0	0.90	0	0	1.00
Veronica peregrina L.	0	0	0	0	0	0	0	0	0	0	0	0	0	0	0	0	0	0
Veronica polita Fr.	0	0	0	0	0	0	0	0	0	0	0	0	0	0	0	0	0	0
Viola sororia Willd.	0	0	0	0	0	0	0	0	0	0	0	0	0	0	0	0	0	0
Zizia aurea (L.) W.D.J.Koch	2.00	0	0	0	0	0	0	0	0	0	0	0	0	0	0	0	0	0
